# Supplementary material for: Credentialing and Pharmacologically Targeting PTP4A3 Phosphatase as a Molecular Target for Ovarian Cancer
Source: Biomolecules. 2021 Jun 30;11(7):969. doi: 10.3390/biom11070969 (PMC8329922; doi:10.3390/biom11070969)
Supplement: Supplementary file 1 [file biomolecules-11-00969-s001.zip › biomolecules-1277442-supplementary.pdf]

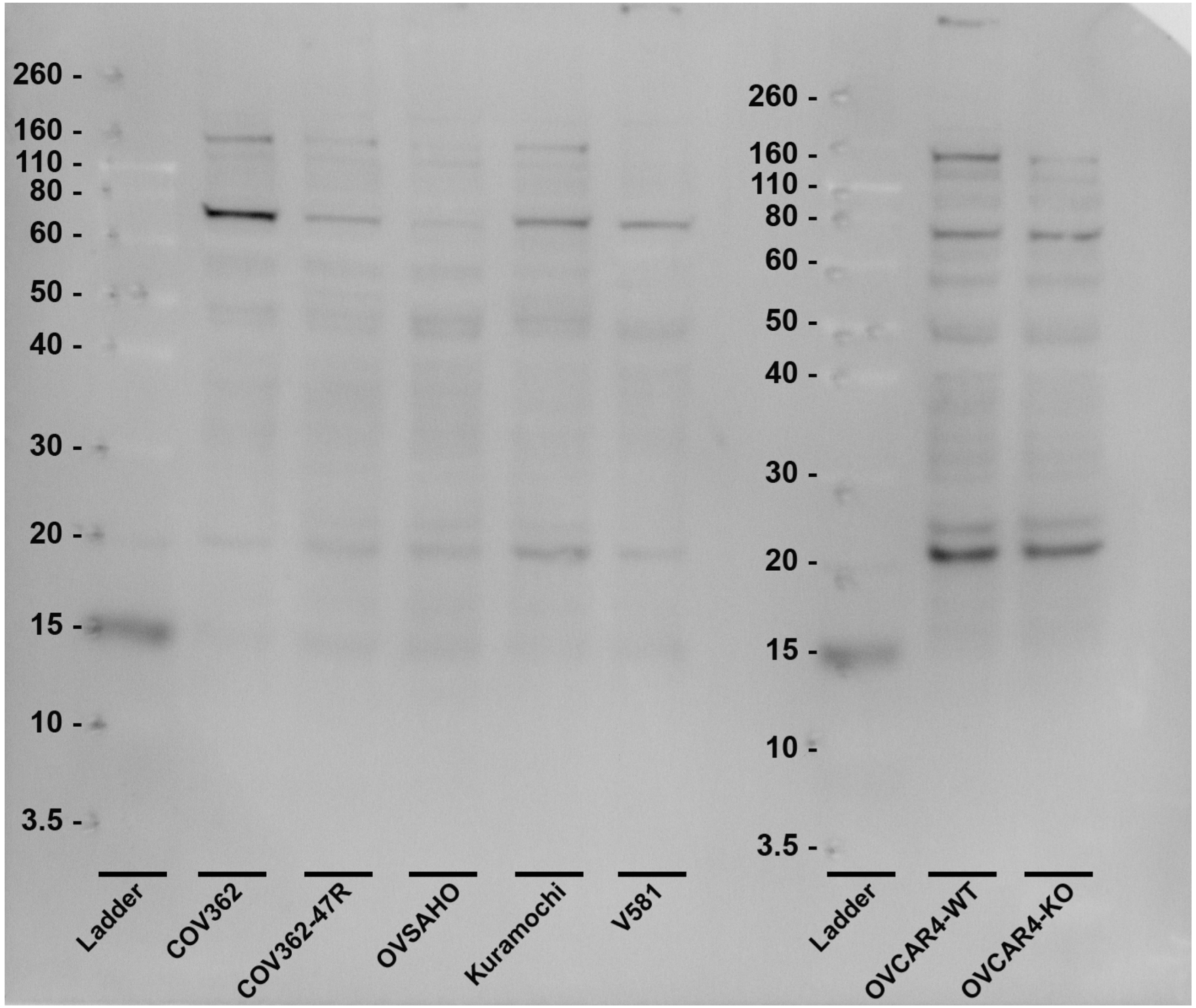

Supplemental Figure S1. Full western blot for PTP4A3 with the Novus antibody. Molecular weight markers are indicated on the left.

Lazo *et al.* Credentialing and Pharmacologically Targeting PTP4A3 Phosphatase as a Molecular Target for Ovarian Cancer.

A.

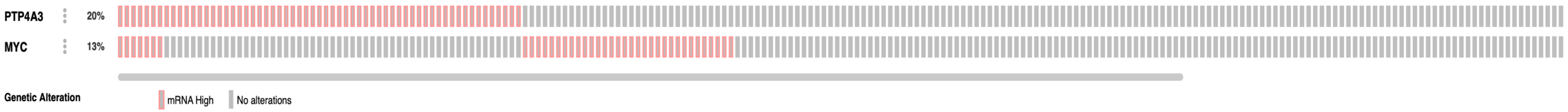

B.

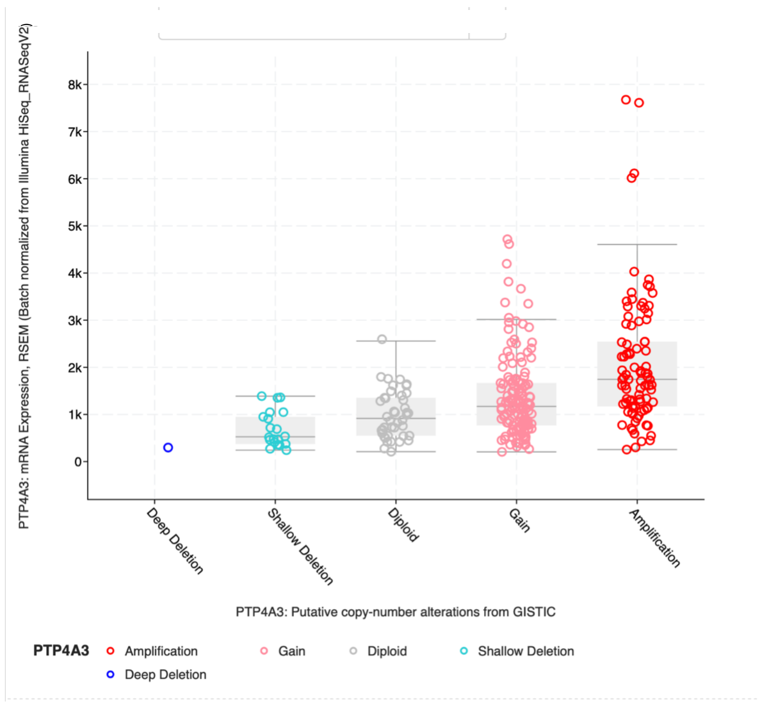

C.

Correlated Gene	Cytoband	Spearman's Correlation	p-Value	q-Value ▲
SLC45A4	8q24.3	0.478	1.61e-18	2.79e-14
MROH6	8q24.3	0.475	2.93e-18	2.79e-14
TSTA3	8q24.3	0.468	9.01e-18	5.71e-14
TSNARE1	8q24.3	0.455	1.02e-16	4.85e-13
SHARPIN	8q24.3	0.449	2.54e-16	9.65e-13
ZFP41	8q24.3	0.448	3.21e-16	1.02e-12
PTK2	8q24.3	0.442	8.77e-16	2.38e-12
ZC3H3	8q24.3	0.438	1.72e-15	4.08e-12
THEM6	8q24.3	0.436	2.47e-15	5.22e-12
LYNX1	8q24.3	0.431	5.14e-15	9.77e-12
ZNF696	8q24.3	0.427	9.82e-15	1.65e-11
HSF1	8q24.3	0.427	1.04e-14	1.65e-11
CHRAC1	8q24.3	0.426	1.17e-14	1.71e-11
TRAPPC9	8q24.3	0.425	1.41e-14	1.91e-11
DGAT1	8q24.3	0.424	1.57e-14	1.99e-11
GRINA	8q24.3	0.423	2.02e-14	2.40e-11
PYCR3	8q24.3	0.420	2.87e-14	3.21e-11
CYHR1	8q24.3	0.416	5.23e-14	5.52e-11
TIGD5	8q24.3	0.415	6.22e-14	5.99e-11
GPAA1	8q24.3	0.415	6.30e-14	5.99e-11
FAM83H	8q24.3	0.414	7.71e-14	6.98e-11
PUF60	8q24.3	0.412	1.05e-13	9.11e-11
RHPN1-AS1	8q24.3	0.409	1.49e-13	1.23e-10
RHPN1	8q24.3	0.408	1.87e-13	1.43e-10
BOP1	8q24.3	0.408	1.88e-13	1.43e-10

Supplemental Figure S2. Gene amplification in TCGA data for HGSOV. Panel A. Expression of PTP4A3 and c-Myc in HGSOV are not coordinately amplified. Panel B. Increase in PTP4A3 copy number in HGSOC from TCGA dataset. Panel C. Genes co-amplified with PTP4A3.

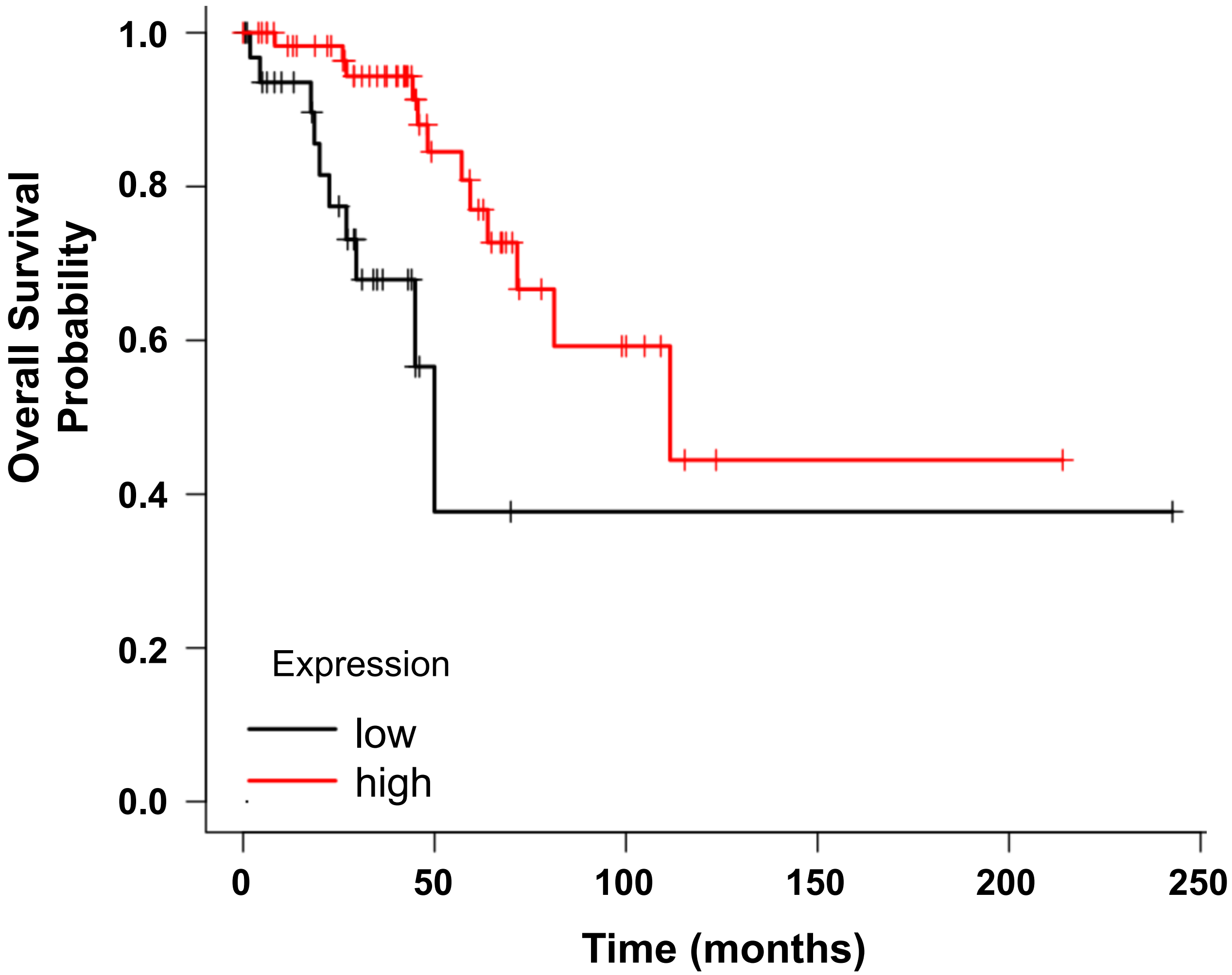

Supplemental Figure S3. High expression of MYC is associated with longer overall survival in patients with stages I/II HGSOC. Data from KMplot 202431_s_at. HR =0.28 (95% Confidence Intervals = 0.12-0.68) from 98 patients with low expressing cohort living 29.6 months and the high expressing cohort living 59.3 months.

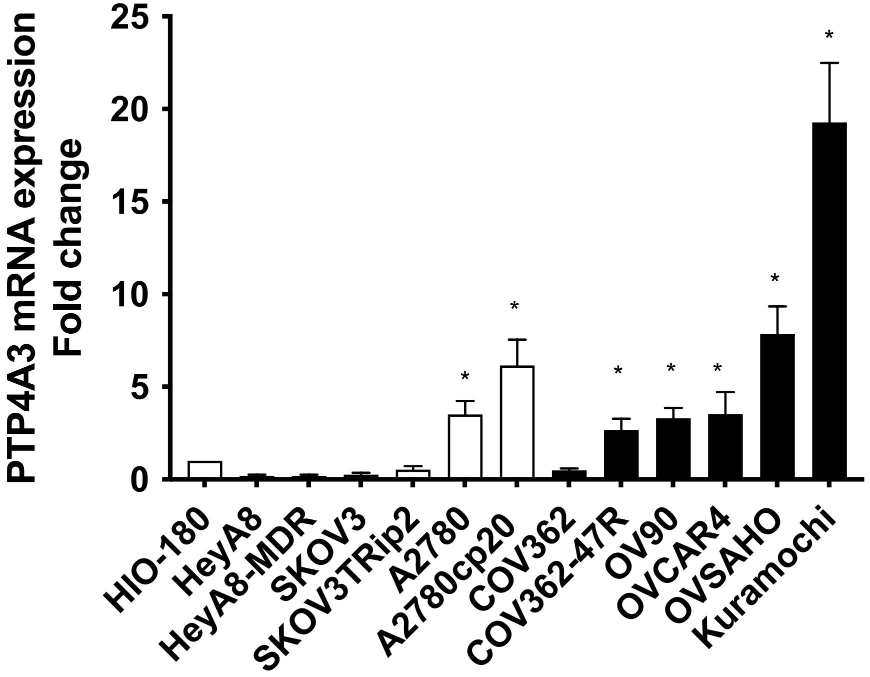

Supplemental Figure S4. PTP4A3 mRNA levels measured by qPCR and normalized to HPRT, actin and GAPDH were elevated in all HGSOC cells lines (black columns) compared to HIO-180 nonmalignant epithelial cells grown in culture with the exception of COV362 cells. * = p<0.05 ANOVA.

Appendix A.

Lazo et al. Credentialing and Pharmacologically Targeting PTP4A3 Phosphatase as a Molecular Target for Ovarian Cancer

A. Chemical Synthesis

1. General Considerations: Unless stated otherwise, all reactions were performed under an atmosphere of N_2 gas that was passed through a column (10 x 2 cm) of Drierite®. Before use, THF was freshly distilled over sodium/benzophenone, and CH_2Cl_2 was freshly distilled over CaH_2. Et_3N and *i*-Pr_2NEt were distilled over CaH_2 and stored over KOH. All glassware and stir bars were dried in an oven for 3 h prior to use. Degassed solvents were obtained by sparging with N_2 for 1 h. Reaction progress was monitored by TLC analysis (pre-coated silica gel 60 F_{254}) and spots were visualized by UV 254 nm and 395 nm). Purifications by chromatography were performed on SiO_2. $^1H/^{13}C$ NMR spectra were recorded on Bruker Avance 300/75 MHz, Bruker Avance 400/100 MHz or Bruker Avance 500/125 MHz instruments. High-resolution mass spectra were obtained on a Micromass UK Limited, Q-TOF Ultima API, or a Thermo Scientific Exactive Orbitrap LC-MS. Chemical shifts were reported in parts per million (ppm) with the residual solvent peak ($CDCl_3$: 7.26 ppm for ^1H, 77.16 ppm for ^{13}C; DMSO-d_6: 2.50 ppm for ^1H, 39.52 ppm for ^{13}C) used as the internal standard. Chemical shifts were tabulated as follows: chemical shift, multiplicity (s = singlet, d = doublet, t = triplet, q = quartet, dd = doublet of doublet, dt = doublet of triplet, ddd = doublet of doublet of doublet, m = multiplet, brs = broad singlet), coupling constant(s), and integration. IR spectra were obtained using neat samples on a Perkin-Elmer 100 ATR-IR spectrometer. Melting points were obtained using

a Mel-Temp instrument and are uncorrected. Photochemical transformations were performed as previously reported.[1,2]

Synthesis of **EJR-912-41** and **EJR-912-43**

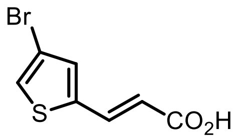

(*E*)-3-(4-Bromothiophen-2-yl)acrylic acid (SI-1). To a solution of thiophene-2-carbaldehyde (2.00 mL, 21.2 mmol) in CH_2Cl_2 (80 mL), $AlCl_3$ (7.19 g, 52.9 mmol) was added. The reaction mixture darkened during the addition and was stirred for 10 min, charged with Br_2 (1.19 mL, 23.3 mmol), and stirred at room temperature for 12 h. The solution was quenched slowly at 0 °C with H_2O and extracted with CH_2Cl_2 (2 × 30 mL). The combined organic layers were washed with saturated aqueous $Na_2S_2O_3$ (30 mL) and saturated aqueous NaCl (30 mL), dried ($MgSO_4$), filtered, and concentrated to give a dark oil. Purification by chromatography on SiO_2 (EtOAc:hexanes, 1:9) gave 4-bromothiophene-2-carboxaldehyde as a light yellow solid (3.43 g) that was used without further characterization.

---

[1] Tasker, N. R., Rastelli, E. J., Blanco, I. K., Burnett, J. C., Sharlow, E. R., Lazo, J. S., and Wipf, P. (2019) In-flow photooxygenation of aminothienopyridinones generates iminopyridinedione PTP4A3 phosphatase inhibitors. *Org Biomol Chem* 17, 2448-2466.

[2] Rastelli, E. J., Yue, D., Millard, C., and Wipf, P. (2021) 3D-Printed cartidge system for in-flow photo-oxygenation of 7-aminotheinopyridinones. *Tetrahedron* **79**, 131875.

A solution of 4-bromothiophene-2-carboxaldehyde (3.43 g, 17.9 mmol) in pyridine (45 mL) was charged with malonic acid (5.66 g, 53.9 mmol) and piperidine (0.89 mL, 8.97 mmol), and heated to reflux. After 3 h, the mixture was concentrated under reduced pressure, diluted with water (100 mL), and a solid precipitated. The mixture was acidified with 6 M HCl until ~pH 2. The precipitate was filtered, washed with water (3 x 20 mL), redissolved in EtOAc (150 mL), dried ($MgSO_4$), filtered, and concentrated to provide a crude residue as an off-white solid. The residue was recrystallized from toluene to provide **SI-1** (1.91 g, 39% over 2 steps) as a white solid: ^{1}H NMR (300 MHz, DMSO-d_6): δ 12.53 (brs, 1 H), 7.81 (d, *J* = 1.2 Hz, 1 H), 7.66 (d, *J* = 15.9 Hz, 1 H), 7.57 (d, *J* = 1.5 Hz, 1 H), 6.26 (d, *J* = 15.9 Hz, 1 H). Spectral data are consistent with literature properties.[3]

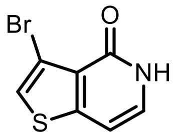

3-Bromothieno[3,2-*c*]pyridin-4(5*H*)-one (SI-2). Triethylamine (1.2 mL, 8.58 mmol) was added to a solution of (*E*)-3-(4-bromothiophen-2-yl)acrylic acid (1.00 g, 4.29 mmol) in acetone (8 mL) at 0 °C. Ethyl chloroformate (1.25 mL, 12.9 mmol) was added, and the reaction mixture was stirred at 0 °C for 30 min before it was slowly charged with a solution of NaN_3 (0.422 g, 6.43 mmol) in H_2O (3.5 mL). The mixture was stirred for 15 min, poured into ice-chilled H_2O, extracted with EtOAc (3 x 40 mL), dried ($MgSO_4$), and concentrated to provide a tan solid that was used without further purification.

[3] Kallepalli, V. A.; Shi, F.; Paul, S.; Onyeozili, E. N.; Maleczka, R. E., Smith III, M. R. *J. Org. Chem*. **2009**, *74*, 3849–3855.

A 3-neck flask fitted with a stopper, addition funnel, and condenser was charged with Bu_3N (1.3 mL, 5.55 mmol), Ph_2O (4.5 mL), and subsequently heated to 240 °C. The addition funnel was charged with a solution of crude acyl azide (1.10 g, 4.26 mmol) in CH_2Cl_2 (12 mL) and added to the reaction mixture over 30 min. This mixture was subsequently stirred at 240 °C for 5 min, cooled to room temperature, and treated with hexanes (5 mL). An orange suspension formed after stirring for 10 min. The precipitate was allowed to settle, hexanes were decanted, and the yellow solid residue was triturated with EtOAc, filtered, and washed with hexanes (3 x 15 mL) to provide **SI-2** (0.449 g, 46% over 2 steps) as a tan solid: Mp >250 °C; IR (ATR): ν_{max} 3102, 3080, 2988, 2940, 2891, 1639, 1598, 1455, 1222, 975, 805, 794, 754, 728 cm^{-1}; ^{1}H NMR (300 MHz, DMSO-d_6): δ 11.47 (brs, 1 H), 7.69 (s, 1 H), 7.29-7.27 (m, 1 H), 6.86 (d, J = 7.2 Hz, 1 H); ^{13}C NMR (125 MHz, DMSO-d_6): δ 157.9, 149.2, 130.8, 124.9, 122.8, 106.9, 101.0; HRMS (ESI$^+$) *m/z:* $[M+H]^+$ calcd for C_7H_5ONSBr 229.9270; Found, 229.9182.

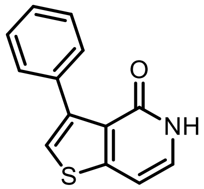

3-Phenylthieno[3,2-*c*]pyridin-4(5*H*)-one (SI-3). In a N_2-filled glove box, a 100-mL flask was charged with $Pd(PPh_3)_4$ (0.200 g, 0.173 mmol) and phenylboronic acid (0.517 g, 4.15 mmol). The flask was removed from the glovebox and sequentially charged with 3-bromothieno[3,2-*c*]pyridin-4(5*H*)-one (0.797 g, 3.46 mmol) and Na_2CO_3 (0.844 g, 7.96 mmol), flushed with N_2, diluted with deoxygenated dioxane/H_2O (2:1, 35 mL), fitted with

a reflux condenser, and heated to 90 °C for 13 h. The resulting orange mixture was cooled to room temperature, diluted with water (150 mL), cooled in an ice-bath, and filtered. The light brown residue was washed with H_2O (2 x 100 mL), dissolved in CH_2Cl_2, dried ($MgSO_4$), and concentrated to give a brown solid that was suspended in CH_2Cl_2 (3 mL). The suspension was filtered to give **SI-3** as a light-yellow solid. The filtrate was concentrated and purified by chromatography on SiO_2 (EtOAc:CH_2Cl_2, 2:3) to provide an additional batch of light-yellow solid **SI-3** (combined amount: 0.640 g, 81%): Mp 187-188 °C; IR (ATR): ν_{max} 2820, 1648, 1610, 1234, 975, 942, 891, 840, 744, 696 cm^{-1}; ^1H NMR (300 MHz, DMSO-d_6): δ 11.30 (brs, 1 H), 7.50-7.46 (m, 3 H), 7.39-7.31 (m, 3 H), 7.26 (t, J = 6.0 Hz, 1 H), 6.87 (d, J = 6.9 Hz, 1H); ^{13}C NMR (100 MHz, DMSO-d_6) δ 158.7, 150.2, 141.1, 135.9, 129.8, 129.6, 127.2, 126.9, 126.0, 123.0, 101.1; HRMS (ESI$^+$) *m/z:* $[M+H]^+$ calcd for $C_{13}H_{10}ONS$ 228.0478; Found 228.0389.

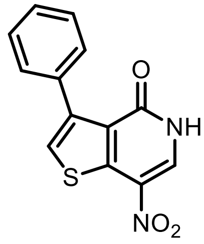

7-Nitro-3-phenylthieno[3,2-*c*]pyridin-4(5*H*)-one (SI-4). A heterogeneous mixture of 3-phenylthieno[3,2-*c*]pyridin-4(5*H*)-one (0.347 g, 1.52 mmol) in MeCN (38 mL) was sparged with O_2 at room temperature for 15 min. Subsequently, *t*-BuONO (0.81 mL, 6.11 mmol) was added and the mixture was stirred under an O_2 atmosphere (1 atm, balloon) for 14 h, concentrated to ~¼ volume, filtered, and the residue was washed with cold MeCN to provide a yellow solid. Purification by flash chromatography on SiO_2 (EtOAc:CH_2Cl_2, 2:3) gave **SI-4** (0.207 g, 50%) as a yellow solid: Mp >250 °C; IR (ATR): ν_{max} 2676, 1649, 1610,

1493, 1341, 1233, 1111, 1021, 995, 764, 745, 696 cm^{-1}; ^{1}H NMR (300 MHz, DMSO-d_6): δ 12.61 (brs, 1 H), 8.74 (s, 1 H), 7.71 (s, 1 H), 7.47-7.35 (m, 5 H); ^{13}C NMR (75 MHz, DMSO-d_6): δ 158.0, 141.2, 141.1, 135.6, 134.9, 129.5, 127.4, 127.3, 127.2, 126.9, 124.6; HRMS (ESI$^+$) *m/z:* [M+H]$^+$ calcd for $C_{13}H_9O_3N_2S$ 273.0328; Found 273.0226.

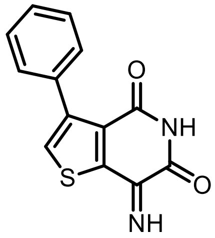

7-Imino-3-phenylthieno[3,2-*c*]pyridine-4,6(5*H*,7*H*)-dione (EJR-912-41). A suspension of 7-nitro-3-phenylthieno[3,2-*c*]pyridin-4(5*H*)-one (**SI-4,** 0.233 g, 0.856 mmol) in dioxane:MeOH (2:1, 20 mL) was treated under an N_2 atmosphere with 10% Pd/C (0.136 g, 0.128 mmol). H_2 was bubbled through the mixture for 10 min, and the reaction mixture was stirred at room temperature under H_2 atmosphere (1 atm, balloon) for 12 h, filtered through Celite, and washed with dioxane/MeOH (1:2, 10 mL portions) until colored bands stopped eluting. The filtrate was concentrated to give a dark solid that was used without further purification in the photo-oxygenation reaction.

A solution of the crude solid in MeOH (850 mL) was passed through the flow apparatus at a rate of 1.9 mL min^{-1} (setting of 5 on the VWR Variable Peristaltic Pump-Model PP3300, corresponding to a 42.0 min residence time) under white LED (40 W) irradiation. The flow apparatus was flushed with MeOH (20 mL). The crude mixture was adsorbed onto Celite and purified by chromatography on SiO_2 (CH_2Cl_2:MeCN, 1:4) to yield the imine/trione mixture as a yellow-orange solid.

The imine/trione mixture was suspended in MeOH (0.009 M) and treated with NH_4OAc (30 eq.) in a sealed pressure tube. The reaction mixture was heated to 60 °C for 18 h before cooling to room temperature and diluting with EtOAc (100 mL). The solution was washed sequentially with saturated aqueous $NaHCO_3$, H_2O, and saturated aqueous NaCl. The combined aqueous washes were extracted with EtOAc (3 x 40 mL or until the aqueous phase was colorless). The combined organic extracts were dried (Na_2SO_4), filtered, and adsorbed onto Celite. Purification by chromatography on SiO_2 ($MeCN:CH_2Cl_2$, 1:4) provided **EJR-912-41** (0.097 g, 44% over 3 steps) as an orange solid: Mp >250 °C; IR (ATR): ν_{max} 3226, 1702, 1611, 1436, 1255, 1212, 1171, 1121, 950, 906, 756, 692 cm^{-1}; ^1H NMR (500 MHz, DMSO-d_6): δ 11.69 (s, 1 H), 11.63 (s, 1 H), 7.88 (s, 1 H), 7.47-7.45 (m, 2 H), 7.42-7.38 (m, 3 H); ^{13}C NMR (75 MHz, DMSO-d_6): δ 159.7, 157.4, 154.2, 145.6, 143.1, 134.5, 131.4, 131.1, 129.3, 127.8, 127.7; HRMS (ESI$^-$) *m/z:* $[M-H]^-$ calcd for $C_{13}H_7O_2N_2S$ 255.0223; Found 255.0230.

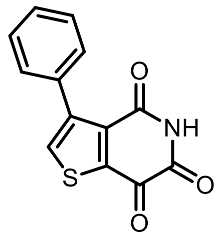

3-Phenylthieno[3,2-*c*]pyridine-4,6,7(5*H*)-trione (EJR-912-43). To a solution of 7-imino-3-phenylthieno[3,2-*c*]pyridine-4,6(5*H*,7*H*)-dione (**EJR-912-41**, 0.052 g, 0.203 mmol) in THF/H_2O (5:1, 5 mL), was added H_2SO_4 (3 drops). The reaction mixture was stirred for 22 h, diluted with EtOAc (50 mL) and washed with saturated aqueous $NaHCO_3$ (3 x 15 mL) and saturated aqueous NaCl (15 mL). The organic layer was dried ($MgSO_4$), filtered, and concentrated to give **EJR-912-43** (0.047 g, 90%) as an orange solid: Mp 256-258 °C;

IR (ATR): ν_{max} 3194, 3097, 1709, 1678, 1436, 1387, 1265, 910, 881, 12, 760, 740, 701, 694, 678 cm^{-1}; ^{1}H NMR (300 MHz, DMSO-d_6): δ 11.75 (brs, 1 H), 8.20 (s, 1 H), 7.48-7.40 (m, 5 H); ^{13}C NMR (125 MHz, DMSO-d_6): δ 169.9, 159.3, 157.6, 143.9, 143.8, 136.3, 135.1, 133.9, 129.3, 128.1, 127.8; HRMS (ESI$^-$) *m/z:* [M-H]$^-$ calcd $C_{13}H_6O_3NS$ 256.0063; Found 256.0071.

<u>Synthesis of **EJR-912-57** and **EJR-912-50**</u>

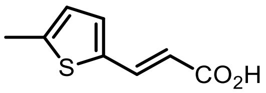

(*E*)-3-(5-methylthiophen-2-yl)acrylic acid (SI-5). A solution of 5-methyl-2-thiophenecarboxaldehyde (4.00 g, 31.1 mmol) in pyridine (77 mL) was treated with malonic acid (9.76 g, 93.2 mmol) and piperidine (1.55 mL, 15.5 mmol) and heated to reflux. After 3 h, the reaction mixture was concentrated under reduced pressure and diluted with water (100 mL) whereupon a solid precipitated. The mixture was acidified with 6 M HCl until ~pH 2. The precipitate was filtered and washed with water (3 x 20 mL). The filter cake was dissolved in EtOAc (150 mL), dried ($MgSO_4$), filtered, and concentrated to provide an off-white solid. The crude material was recrystallized from toluene to provide **SI-5** (3.21 g, 61%) as a white solid: ^{1}H NMR (300 MHz, $CDCl_3$): δ 7.79

(d, J = 15.6 Hz, 1 H), 7.09 (d, J = 3.6 Hz, 1 H), 6.73 (dd, J = 3.6 Hz, 0.9 Hz, 1 H), 6.09 (d, J = 15.6 Hz, 1 H), 2.51 (s, 3 H). Spectral data are consistent with literature values.[4]

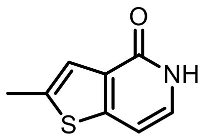

2-Methylthieno[3,2-*c*]pyridin-4(5*H*)-one (SI-6). Triethylamine (5.36 mL, 38.2 mmol) was added to a solution of (*E*)-3-(5-methylthiophen-2-yl)acrylic acid (**SI-5**, 3.21 g, 19.1 mmol) in acetone (36 mL) at 0 °C (ice-bath). Subsequently, ethyl chloroformate (5.58 mL, 57.3 mmol) was added, and the mixture was stirred at 0 °C for 1.5 h. A solution of NaN_3 (1.88 g, 28.6 mmol) in H_2O (14 mL) was slowly added to the reaction mixture at 0 °C. The mixture became homogeneous and then a solid began to precipitate. Stirring continued for 15 min. The reaction mixture was poured into ice-chilled H_2O (30 mL) and extracted with EtOAc (3 x 30 mL). The combined organic layers were dried ($MgSO_4$), filtered and concentrated to give crude acyl azide as a tan solid that was used for the next step without further purification.

A 3-neck flask fitted with a stopper, addition funnel, and condenser was charged with Bu_3N (5.89 mL, 24.8 mmol) and Ph_2O (19 mL), and was subsequently heated to 240 °C. The addition funnel was charged with a solution of the crude acyl azide (3.68 g, 19.0 mmol) in CH_2Cl_2 (30 mL). The acyl azide solution was added slowly to the hot reaction mixture over ca. 40 min. to allow the CH_2Cl_2 to boil off. The mixture was stirred at 240 °C

---

[4] Yang, B.; Vasbinder, M. M.; Hird, A. W.; Su, Q.; Wang, H. ; Yu, Y.; Toader, D.; Lyne, P. D.; Read, J. A.; Breed, J.; Ioannidis, S.; Deng, C.; Grondine, M.; DeGrace, N.; Whitston, D.; Brassil, P.; Janetka, W. W. *J. Med. Chem*. **2018**, *61*, 1061-1073. *J. Org. Chem*. **2009**, *74*, 3849–3855

for an additional 15 min. Upon cooling to room temperature, hexanes (20 mL) were added, and a solid began to precipitate. The hexanes layer was decanted, and the remaining residue was suspended in EtOAc (15 mL). The solid precipitate was filtered to provide **SI-6** (2.78 g, 88% over 2 steps) as a tan solid: Mp 214-215 °C; IR (ATR): ν_{max} 2818, 1648, 1604, 1275, 1225, 1124, 1076, 960, 765 cm^{-1}; ^{1}H NMR (300 MHz, DMSO-d_6): δ 11.30 (brs, 1 H), 7.17-7.15 (m, 2 H), 6.73 (d, *J* = 6.9 Hz, 1 H), 2.50 (s, 3 H, overlapping the DMSO signal); ^{13}C NMR (125 MHz, DMSO-d_6): δ 158.3, 147.3, 138.4, 130.7, 128.8, 121.8, 100.8, 15.1; HRMS (ESI$^+$) *m/z:* $[M+H]^+$ calcd for C_8H_8NOS 166.0321; Found 166.0313.

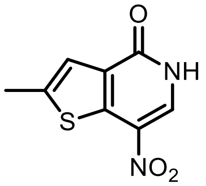

2-Methyl-7-nitrothieno[3,2-*c*]pyridin-4(5*H*)-one (SI-7). A solution of 2-methylthieno[3,2-*c*]pyridin-4(5*H*)-one (**SI-6**, 2.25 g, 13.6 mmol) in MeCN (340 mL) at room temperature was fitted with an O_2 balloon (1 atm), and O_2 was bubbled through the solvent for 15 min. Subsequently, *t*-BuONO (7.19 mL, 54.5 mmol) was added, and the mixture was stirred under an O_2 atmosphere (1 atm, balloon) at 60 °C for 3 h, concentrated to ~ ¼ volume, and the resulting suspension was placed in a -20 °C freezer for 2 h before filtering and washing with cold MeCN to provide **SI-7** (1.90 g, 66%) as a yellow solid: Mp >250 °C; IR (ATR): ν_{max} 2832,1649, 1613, 1496, 1339, 1248, 1231, 1118, 1040, 897, 840, 825, 767, 707 cm^{-1}; ^{1}H NMR (500 MHz, DMSO-d_6): δ 12.9 (brs, 1 H), 8.63 (s, 1 H(m 7.27 (d, *J* = 1.0 Hz, 1 H), 2.54 (d, *J* = 1.0 Hz, 3 H); ^{13}C NMR (75 MHz, DMSO-d_6): δ 157.7,

142.4, 137.9, 134.4, 129.1, 127.3, 121.9, 14.7; HRMS (ESI$^+$) *m/z:* [M+H]$^+$ calcd for $C_8H_7O_3N_2S$ 211.0171; Found 211.0161.

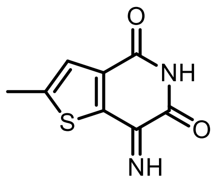

7-Imino-2-methylthieno[3,2-*c*]pyridine-4,6(5*H*,7*H*)-dione (EJR-912-57). A suspension of 2-methyl-7-nitrothieno[3,2-*c*]pyridin-4(5*H*)-one (**SI-7**, 0.250 g, 1.19 mmol) in dioxane:MeOH (2:1, 30 mL) was treated under an N_2 atmosphere with 10% Pd/C (0.190 g, 0.178 mmol). H_2 was bubbled through the mixture for 10 min. The suspension was stirred at rt under H_2 (1 atm, balloon) for 12 h, filtered through Celite, and washed with dioxane:MeOH (1:2, 10 mL portions) until colored bands stopped eluting. The filtrate was concentrated to give a dark solid that was used without further purification in the photo-oxygenation reaction.

A solution of the crude solid in MeOH (800 mL) was passed through the flow apparatus at a rate of 1.9 mL min^{-1} (setting of 5 on the VWR Variable Peristaltic Pump-Model PP3300, corresponding to a 42.0 min residence time) under white LED (40 W) irradiation. The tubing was flushed with MeOH (20 mL). The mixture was adsorbed onto Celite and purified by chromatography on SiO_2 (CH_2Cl_2:MeCN) to yield the a mixture of imine/trione as a yellow-orange solid.

The imine/trione mixture was suspended in MeOH (0.009 M) and treated with NH_4OAc (30 eq.) in a sealed pressure tube. The reaction mixture was heated to 60 °C for 18 h before cooling to room temperature and diluting with EtOAc (100 mL). The solution was

washed sequentially with saturated aqueous $NaHCO_3$, H_2O, and saturated aqueous NaCl. The combined aqueous washes were extracted with EtOAc (3 x 40 mL or until the aqueous phase was colorless). The combined organic extracts were dried (Na_2SO_4), filtered, and concentrated to give **SI-7** (0.101 g, 44% over 3 steps) as a brown solid: Mp > 250 °C; IR (ATR): ν_{max} 3010, 2829, 1707, 1685, 1617, 1474, 1425, 1382, 1307, 1244, 1169, 914, 867, 774 cm^{-1}; ^1H NMR (300 MHz, DMSO-d_6): δ 11.46-11.41 (brs, 2 H), 7.29 (d, J = 1.2 Hz, 1 H), 2.56 (d, J = 1.2 Hz, 3 H); ^{13}C NMR (125 MHz, DMSO-d_6): δ 160.0, 157.9, 153.2, 147.5, 141.4, 135.8, 124.5, 15.5; HRMS (ESI$^+$) *m/z:* $[M+H]^+$ calcd for $C_8H_7O_2N_2S$ 195.0223; Found 195.0223.

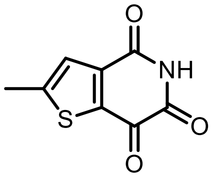

2-Methylthieno[3,2-*c*]pyridine-4,6,7(5*H*)-trione (EJR-912-50). A solution of 7-imino-2-methylthieno[3,2-*c*]pyridine-4,6(5*H*,7*H*)-dione (**EJR-912-57**, 0.045 g, 0.231 mmol) in THF:H_2O (5:1, 6 mL) was treated with H_2SO_4 (3 drops), stirred for 16 h, diluted with EtOAc (50 mL) and washed with saturated aqueous $NaHCO_3$ (3 x 15 mL) and saturated aqueous NaCl (15 mL). The organic layer was dried ($MgSO_4$), filtered, and concentrated to provide **EJR-912-50** (0.020 g, 44%) as an orange solid: Mp > 191 °C (dec); IR (ATR): ν_{max} 3073, 2925, 2856, 1730, 1667, 1456, 1438, 1419, 1291, 1225, 1184, 879, 829 cm^{-1}; ^1H NMR (300 MHz, DMSO-d_6): δ 11.80 (brs, 1 H), 7.40 (d, J = 1.0 Hz, 1 H), 2.62 (d, J = 1.0 Hz, 3 H); ^{13}C NMR (125 MHz, DMSO-d_6): δ 168.5, 159.6, 158.1, 153.7, 140.3, 139.6, 125.9, 15.9; HRMS (ESI$^+$) *m/z:* $[M+H]^+$ calcd for $C_8H_6O_3NS$ 196.0063; Found 196.0062.

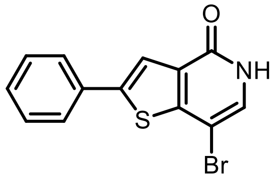

7-Bromo-2-phenylthieno[3,2-*c*]pyridin-4(5*H*)-one (NRT-902-38). A solution of 2-phenylthieno[3,2-c]pyridin-4(5*H*)-one[1] (0.500 g, 2.20 mmol) in AcOH (6.3 mL) was treated at room temperature with Br_2 (0.136 mL, 2.64 mmol), heated at reflux for 30 min, cooled to room temperature, and treated with H_2O (6 mL). A precipitate formed that was filtered, washed with H_2O (2 x 5 mL), and dried to give **NRT-902-38** (0.547 g, 81%) as a brown solid: Mp 249-251 °C; IR (ATR): ν_{max} 3049, 2776, 1651, 1596, 1532, 1499, 1482, 1445, 1362, 1331, 1273, 1235, 1206, 1182, 1142, 1093, 1069, 1030, 984, 838, 748, 707, 681 cm^{-1}; ^{1}H NMR (400 MHz, DMSO-d_6): δ 11.81 (brs, 1 H), 8.01 (s, 1 H), 7.81 (d, *J* = 1.2 Hz, 2 H), 7.59 (s, 1 H), 7.47 (t, *J* = 8.0 Hz, 2 H), 7.38 (td, *J* = 5.6, 8.4 Hz, 1 H); ^{13}C NMR (100 MHz, DMSO-d_6) : δ 157.6, 148.5, 142.2, 132.5, 131.2, 130.5, 129.3, 128.7, 125.8, 121.2, 91.5; HRMS (ESI$^+$) *m/z:* $[M+H]^+$ calcd for $C_{13}H_9BrNOS$ 307.9555; Found 307.9556.
